# Psyllium reduces inulin-induced colonic gas production in IBS: MRI and *in vitro* fermentation studies

**DOI:** 10.1136/gutjnl-2021-324784

**Published:** 2021-08-05

**Authors:** David Gunn, Zainab Abbas, Hannah C Harris, Giles Major, Caroline Hoad, Penny Gowland, Luca Marciani, Samantha K Gill, Fred J Warren, Megan Rossi, Jose Maria Remes-Troche, Kevin Whelan, Robin C Spiller

**Affiliations:** 1 NIHR Nottingham Biomedical Research Centre, Nottingham University Hospitals NHS Trust and the University of Nottingham, Nottingham, UK; 2 Nottingham Digestive Diseases Centre, University of Nottingham, Nottingham, UK; 3 Food, Innovation and Health, Quadram Institute Bioscience, Norwich, UK; 4 Sir Peter Mansfield Imaging Centre, University of Nottingham, Nottingham, UK; 5 Department of Nutritional Sciences, King's College London, London, UK; 6 Medical Biological Research Institute, Veracruzana University, Veracruz, Mexico

**Keywords:** irritable bowel syndrome, colonic fermentation, abdominal MRI, dietary fibre

## Abstract

**Objective:**

Health-promoting dietary fibre including inulin often triggers gastrointestinal symptoms in patients with IBS, limiting their intake. Our aim was to test if coadministering psyllium with inulin would reduce gas production.

**Design:**

A randomised, four-period, four-treatment, placebo-controlled, crossover trial in 19 patients with IBS. Subjects ingested a 500 mL test drink containing either inulin 20 g, psyllium 20 g, inulin 20 g+ psyllium 20 g or dextrose 20 g (placebo). Breath hydrogen was measured every 30 min with MRI scans hourly for 6 hours. Faecal samples from a subset of the patients with IBS were tested using an *in vitro* fermentation model. Primary endpoint was colonic gas assessed by MRI.

**Results:**

Colonic gas rose steadily from 0 to 6 hours, with inulin causing the greatest rise, median (IQR) AUC_(0–360 min)_ 3145 (848–6502) mL·min. This was significantly reduced with inulin and psyllium coadministration to 618 (62–2345) mL·min (p=0.02), not significantly different from placebo. Colonic volumes AUC_(0–360 min)_ were significantly larger than placebo for both inulin (p=0.002) and inulin and psyllium coadministration (p=0.005). Breath hydrogen rose significantly from 120 min after inulin but not psyllium; coadministration of psyllium with inulin delayed and reduced the maximum increase, AUC_(0–360 min)_ from 7230 (3255–17910) ppm·hour to 1035 (360–4320) ppm·hour, p=0.007.

Fermentation *in vitro* produced more gas with inulin than psyllium. Combining psyllium with inulin did not reduce gas production.

**Conclusions:**

Psyllium reduced inulin-related gas production in patients with IBS but does not directly inhibit fermentation. Whether coadministration with psyllium increases the tolerability of prebiotics in IBS warrants further study.

**Trial registration number:**

NCT03265002.

**Significance of this study**

**What is already known on this subject?**

- Low-fermentable oligo-saccharide, di-saccharide, mono-saccharide and polyols (FODMAP) diets not only reduce IBS symptoms but also alter the gut microbiome reducing *Bifidobacteria* and other beneficial bacteria.
- Ingestion of inulin, a common dietary fibre, increases colonic gas and breath hydrogen.
- Psyllium has been shown in randomised placebo-controlled trials to reduce IBS symptoms.

**What are the new findings?**

- After studying patients with IBS we have shown that
  - Psyllium produces an increase in colonic volumes without increasing colonic gas or breath hydrogen.
  - Psyllium is only slowly fermented by IBS faecal microbiota.
  - Combining psyllium with inulin reduces both colonic gas and breath hydrogen response in patients with IBS but *in vitro* does not impair fermentation.

**How might it impact on clinical practice in the foreseeable future?**

- Our findings suggest that by taking viscous, poorly fermented fibres such as psyllium when FODMAP-rich foods are eaten, it may be possible to achieve the reduction of colonic gas symptoms seen on a low-FODMAP diet without disturbing the microbiota or requiring severe dietary restriction.

## Introduction

Patients with IBS often report an unpleasant awareness of intestinal gas associated with a sensation of abdominal distension, commonly described as bloating. The connection between intestinal gas and gut symptoms is complex as signalling from gut to brain is influenced by many factors in patients with IBS including mood and somatisation.^1^ Although cross-sectional studies of unselected patients with IBS have not shown excessive intestinal gas compared with healthy controls,^2^ increases in gas have been related to symptom induction possibly related to visceral hypersensitivity.^3^ Inulin-type fructans worsen some symptoms in IBS particularly when given at higher doses.^4^ Despite the apparent clinical benefit of a low-fermentable oligoaccharide, diaccharide, monosaccharide and polyols (FODMAP) diet,^5^ it inevitably reduces intake of substances which act as prebiotics, leading to a reduction in key bacterial genera in the gut including *Bifidobacteria*,^6 7^ with potential negative consequences.^8^ Alternative approaches to control of colonic fermentation in IBS are therefore being actively pursued.

Viscous fibres such as psyllium, an arabinoxylan polymer that resists digestion in the human upper gastrointestinal tract,^9^ improve symptoms in unselected IBS^10^ though until now the mechanism was unclear. We have recently used MRI to show that psyllium’s open network of polymers traps water in the small bowel and increases ascending and descending colon water content. We believe that this is the basis of its laxative effect, softening stool so that it is easier to pass.^11^ However, the small bowel effects may also be important since psyllium forms a highly viscous solution in the upper small intestine which interferes with absorption.^12^ Psyllium is only slowly fermented so its viscosity effects are likely to persist for some hours after it reaches the colon. We have previously used radio-isotopic imaging to demonstrate that psyllium 3.5 g three times a day substantially reduced lactulose-associated acceleration of proximal colonic transit in healthy volunteers, but limitations of the method used means the mechanisms of action was unclear.^13^

The current study uses MRI to test the hypothesis that psyllium can reduce the increase in colonic gas induced in IBS by inulin. Inulin is a macropolymer which reaches the colon largely intact. There it is rapidly fermented producing large amounts of gas^14^ which correlates with symptoms of flatulence, bloating, discomfort and pain in patients with IBS.^3^ We further investigated the potential mechanisms of action by assessing the viscosity of test substances, known to affect fibre functionality,^15 16^ and their *in vitro* gas production by microbiota derived from stool donated from the participants with IBS.

## Materials and methods

Two studies were performed, both investigating the effect of psyllium and inulin, both alone and when coadministered, on gas production in IBS. An *in vivo* study used MRI and breath hydrogen in patients with IBS and an *in vitro* study measured the viscosity of test solutions and their fermentation in a laboratory model of colonic fermentation using stool samples from participants in the *in vivo* study.

The carbohydrates used were inulin (Orafti HP, Beneo, Mannheim, Germany) which is 99.9% pure with average degree of polymerisation >23 and psyllium husk (Supernutrients, Bath, UK) which is 88.9% dietary fibre with 23.5% being soluble,^17^ degree of polymerisation being >2800.^18^ The placebo used was food grade dextrose (Thornton & Ross, Huddersfield, UK).

### Human MRI study

This was a single-centre, four-period, four-treatment, placebo-controlled, crossover trial. Each treatment was administered once to each participant, with randomisation of treatment order. The treatments were test drinks containing inulin, psyllium, inulin and psyllium and dextrose as a control.

#### Participants

Participants were recruited through the digestive diseases services at Nottingham University Hospitals NHS Trust and the Circle Treatment Centre, through primary care via the NIHR Clinical Research Network in England and through general advertisement. Eligible participants were aged over 16 years, fulfilling Rome IV criteria^19^ for either diarrhoea-predominant or constipation-predominant IBS. Exclusion criteria included: pregnancy, contraindications to MRI scanning, inability to cease use of supplementary fibres or laxatives, inability to stop drugs affecting GI motility, history of other pre-existing gastrointestinal disorders or resection, intention to change smoking habit during the study, excess alcohol intake or anyone who in the opinion of the investigator would be unable to comply with the study protocol.

#### Test drinks and controlled diet

Participants were provided with a low-fibre, low-FODMAP meal to eat at home the evening prior to and during the study day in order to standardise intake of foods likely to increase colonic gas (see online supplemental tables S1A, B for nutritional information).

10.1136/gutjnl-2021-324784.supp1 Supplementary data

The test drinks contained (a) 20 g inulin powder, (b) 20 g psyllium powder, (c) 20 g inulin powder and 20 g psyllium powder and (d) 20 g dextrose powder as a placebo control. Inulin and dextrose were dissolved in boiling water which was subsequently cooled in a refrigerator overnight. Test drinks containing psyllium were divided into four 125 mL drinks with a quarter of the psyllium added to each just prior to ingestion to avoid gelling which gave the solutions an unpleasant texture. The other drinks were imbibed at the same rate so that the 500 mL total for all test substances was consumed over 5 min. All test substances were stored in a sealed container in a cool, dry and dark environment prior to consumption.

#### Protocol

Participants were provided with written information about the study at least 24 hours prior to informed consent. Eligibility was confirmed and information including medical history, current medications, height, weight and smoking history was recorded. Their IBS subtype was confirmed according to Rome IV criteria using a 1-week stool diary documenting stool frequency and consistency on the Bristol Stool Form Scale (BSFS).^19^ Participants completed the Hospital Anxiety and Depression Scale^20^ and the Patient Health Questionnaire 12 Somatic Symptom Scale^21^ questionnaires. Eligible participants were randomised to the order of test drink intake using www.randomization.com and given a dietary advice sheet for the day preceding each study day. Participants were provided with a low-fibre, low-FODMAP evening meal to eat at home before 20:00 on the evening prior to MRI scans (online supplemental table S1A).

Participants fasted from 20:00 the day prior to the study day and were allowed only sips of water for essential tablets as necessary. On the morning of the study they attended the University of Nottingham Sir Peter Mansfield Imaging Centre and underwent a fasted MRI scan on a 1.5 T GE MRI scanner using a parallel imaging 12-element torso coil (see online supplemental material MRI endpoints and methods for details). They also had breath hydrogen measured by exhaling into a gas analyser (GastroCH4ECK, Bedfont, UK) and recorded symptoms of flatulence, bloating and abdominal pain using a modified Gastrointestinal Symptom Rating Scale.^22^ This asks subjects to rate their symptoms on a 7-point scale 0–3 with intervals of 0.5 units, with anchors being 0=none, 1=mild, 2=moderate and 3=severe. Participants then consumed the allocated test drink. MRI scans were performed immediately after the drink and then hourly for 6 hours, while hydrogen breath tests and symptom scoring occurred every half hour (see online supplemental figure S1 for study day details). After 210 min, participants received a 338 kcal meal of rice pudding, jam and orange juice (see online supplemental table S1B for nutritional information) and completed a hydrogen breath test and symptom score. Participants spent approximately 15 min inside the MRI at any one time. A washout period of at least 6 days was used between study days. Subjects collected their own stool samples at home using a dedicated stool collection kit (see online supplemental material for details). Samples were double bagged and frozen at −20°C in a domestic freezer before being brought to the unit in an insulated bag on an ice pack to avoid thawing. Samples were consecutive samples requested from all subjects and were thus a random sample with no selection criteria.

The primary endpoint was the change from fasting values in colonic gas assessed from the area under the curve (AUC) from 0 to 360 min in arbitrary units with secondary endpoints being colonic volume AUC (L·min) and small bowel water content (SBWC) AUC (L·min), all measured by MRI. Other secondary endpoints included breath hydrogen in parts per million and severity of pain, bloating and flatulence, each scored 0–3.

### In vitro fermentation study

Gas production from the fermentation of the test substrates was measured using the ANKOM RF gas production system (ANKOM Technology, Macedon, New York, USA).

Fermentation bottles were seeded with faecal samples from eight of the individuals with IBS from the human MRI study (four constipation-predominant IBS (IBS-C), four diarrhoea-predominant IBS (IBS-D)) as previously described.^17^ Faecal samples were frozen at −80°C and defrosted at room temperature prior to testing. Test substance samples were diluted 1 in 10 in prereduced phosphate-buffered saline (10% wt/vol), homogenised in a stomacher and strained to remove particulates. Fermentation bottles containing dextrose (0.5 g acting as a control of known fermentability), psyllium (0.5 g), inulin (0.5 g) or both inulin and psyllium (0.5 g+0.5 g) were inoculated with 3 mL of slurry, sealed so anaerobic and incubated at 37°C in a shaking water bath (80 rpm) for 5 days. Gas pressure was automatically measured every 15 min for 48 hours using the ANKOM RF system. Gas production from fibre was calculated using previous methods.^23^ Data are reported as cumulative gas volume produced during fermentation. Further technical details are given in online supplemental material.

#### Viscosity assessment

Substrate viscosity was assessed using an AR-G2 rheometer (TA Instruments, Delaware, USA) using a cup and vane geometry. Analysis was performed at the same concentration as predicted to occur *in vivo* (4% solution assuming 20 g is distributed in a colonic volume of around 500 mL) and *in vitro* (0.5% solution). Inulin was solubilised in boiling H_2_0 and stored at 4°C overnight. Psyllium was mixed with water or the inulin solution immediately prior to analysis at a frequency of 6.28 rad/s (1 Hz). The frequency was determined using a strain-sweep experiment to be within the linear viscoelastic range.

### Statistical analysis

The sample size was calculated using data from a previous study of the effect of inulin on colonic gas in IBS.^3^ To detect an increase of 40 mL of gas at 80% power and alpha of 1.66%, we calculated 20 participants would be required to complete the study.^24^ We planned to enrol up to 25 participants to allow for 20% dropout. We considered 40 mL to be the minimally clinically important difference, representing approximately a 20% increase in ascending colon volume. All statistical analysis was performed using GraphPad Prism V.8.2.1 or later for Windows (GraphPad Software, La Jolla, California, USA). Normally distributed data are presented as mean±SD and non-normally distributed data as median (IQR) and analysed using parametric and non-parametric statistical tests, respectively.

Profiles of values over time are summarised as AUC from 0 to 360 min for the MRI study and from 0 to 48 hours for the *in vitro* fermentation studies. Differences between the AUC of the change from fasting values for MRI colonic gas, AUC for breath hydrogen and for *in vitro* gas production were compared for the four test carbohydrates using the non-parametric Friedman test with Dunn’s multiple comparisons as a post hoc test. The differences in colonic volume AUC, SBWC AUC and patient symptoms at 6 hours were normally distributed and analysed using repeated measures one-way analysis of variance (RM ANOVA) with Tukey’s multiple comparisons as the post hoc test. Comparisons of data between the two IBS subtypes were performed using Mann-Whitney test.

Gas production from inulin and psyllium coadministration was measured in the *in vitro* fermentation studies and theoretical values were calculated by summation of measured values for inulin alone and psyllium alone. Calculated inulin and psyllium was treated as an additional variable for all analyses. Comparisons of *in vitro* data between IBS subtype per substrate were performed using Mann-Whitney test.

MRI images were anonymised to ensure the analysis was done blind as to the treatment. The methods used were as previously published for SBWC,^25^ colonic gas^26^ and colonic volumes^27^ (see online supplemental material).

### Patient and public involvement (PPI)

Patients were not involved in the details of the study design and endpoints; however, these were very similar to previous studies^3^ which had benefited from PPI.

## Results

### Human MRI study participants

Overall, 26 patients attended the initial screening visit, of which 19 completed the study (for Consolidated Standards of Reporting Trials diagram see online supplemental figure S2). Overall, 15 patients were female and 4 were male, with a median age of 39 (min–max 19–65) years. Overall, 9 participants suffered from constipation-predominant IBS (IBS-C) and 10 from diarrhoea-predominant IBS (IBS-D). There were no significant differences in age, gender, anxiety or depression scores between IBS-C and IBS-D (table 1). Patients with IBS-D had a higher body mass index (BMI) (35.0±7.1 kg/m^2^ vs 26.2±5.7 kg/m^2^, p=0.009) and as expected had a greater frequency of bowel movements (p*=*0.02) and higher BSFS Score (p=0.02) than patients with IBS-C. All tolerated the study procedures well.

**Table 1:** Characteristics of study participants by IBS subtype

Characteristic	Constipation-predominant IBS (n=9)	Diarrhoea-predominant IBS (n=10)	P value
Age, median (min–max)	39 (19–65)	43 (19–65)	0.92
Gender, male/female	2/7	2/8	>0.99
BMI (kg/m^2^), mean±SD	26.2±5.7	35.0±7.1	0.009
HADS-A, mean±SD	6.8±6.3	5.6±3.7	0.62
HADS-D, mean±SD	2.8±3.1	3.6±2.4	0.34
PHQ-12SS, mean±SD	4.6±4.4	7.7±3.4	0.10
Weekly bowel frequency, mean±SD	7.7±3.0	12.4±5.0	0.02
Weekly Bristol Stool Form Scale Score, mean±SD	2.1±0.8	5.3±0.8	0.02

BMI, body mass index; HADS-A, Hospital Anxiety and Depression Scale-Anxiety subscale; HADS-D, Hospital Anxiety and Depression Scale-Depression subscale; PHQ-12SS, Patient Health Questionnaire-12 Somatic Symptom Scale.

#### Colonic gas and colonic volume

**Figure 1:**
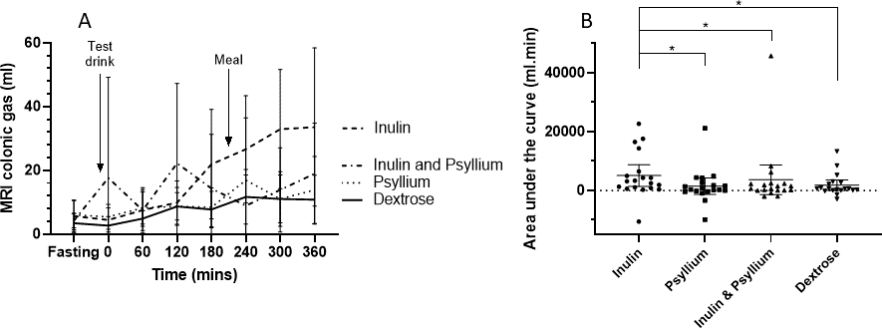
Change in MRI colonic gas from fasting values for each test drink (n=19). Data shown are mean±95% CI. (A) Time course over the duration of the study, showing significantly greater gas production for inulin compared with psyllium, dextrose and inulin and psyllium coadministration at 360 min (p=0.0097). (B) Area under the curve (AUC) for individual participants after each test drink (on the x axis). Inulin produced a significantly larger AUC than the other three test drinks. *p<0.05.

Colonic gas, as measured by MRI, rose steadily during the course of the study period, reaching a maximum at 360 min (figure 1A). The AUC of the change in colonic gas from fasting to 360 min (AUC gas, the primary outcome) was significantly affected by the fibre given (figure 1B) (Friedman test, p=0.005). Inulin caused the greatest rise in colonic gas (AUC gas 3145 (848–6502) mL·min) which was significantly reduced by the coadministration of inulin and psyllium (618 (62–2345) mL·min, p=0.02), the latter not being significantly different from dextrose (p>0.99).

**Figure 2:**
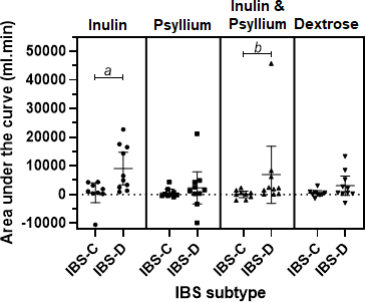
Area under the curve (AUC) change in MRI colonic gas from fasting values for IBS-C versus IBS-D (n=19), demonstrating significantly greater AUCs for IBS-D after both inulin and inulin and psyllium coadministration test drinks. Data shown are mean±95% CI. a, p=0.01. b, p=0.03. IBS-C, constipation-predominant IBS; IBS-D, diarrhoea-predominant IBS.

IBS subtype influenced the AUC gas which was larger in IBS-D compared with those with IBS-C for both inulin (Mann-Whitney test, p=0.01) and inulin with psyllium coadministration (p=0.03), but not for dextrose or psyllium alone (figure 2 and online supplemental table S2).

**Figure 3:**
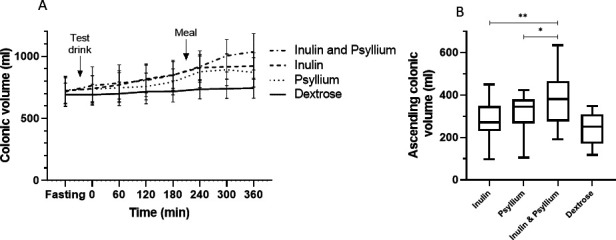
Colonic volumes after the test drink (n=19). (A) Total colonic volumes after dextrose remained stable but rose significantly after the other three test drinks. Area under the curve for both inulin and inulin and psyllium coadministration were significantly greater than dextrose, p=0.002 and p=0.005, respectively. Data shown are mean±95% CI. (B) Ascending colonic volume 360 min after each test drink (on the x axis), represented by Tukey box and whiskers plot. Inulin and psyllium coadministration significantly increased the volume compared with either inulin or psyllium alone (p=0.003 and p=0.02, respectively).

Total colonic volume remained unaltered by dextrose, rose steadily for both psyllium alone and inulin alone but the greatest rise occurred with inulin and psyllium coadministration (figure 3A). RM ANOVA showed a significant difference between test drinks (p=0.01); post hoc analyses showed greater AUCs for inulin (353.8±85.0 L·min) and inulin and psyllium coadministration (362.5±109.0 L·min) compared with dextrose (300.2±73.0 L·min, p=0.002 and p=0.005), respectively. Since the effect of increased small bowel water inflow would be maximal in the ascending colon, we also measured just the ascending colon at 360 min when the differences between the test drinks were highly significant (p=0.0016). Coadministration of inulin and psyllium increased ascending colon volume at 360 min compared with inulin alone or psyllium alone (p=0.0026 and p=0.018, respectively, figure 3B, see online supplemental table S3 for regional AUCs). The differences in regional AUCs between the test drinks are most obvious proximally as the test material would not have progressed beyond the transverse colon during the 6-hour study period. Unlike colonic gas however, there were no differences in colonic volumes in response to the test drinks between IBS subtypes (online supplemental table S2).

#### Breath hydrogen

**Figure 4:**
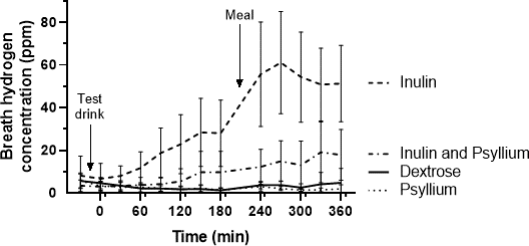
Breath hydrogen concentration (ppm) at fasting and every 30 min after the test drink (n=19). Breath hydrogen rose steadily after 30 min with the inulin test drink but area under the curve analysis demonstrated a significantly reduced rise when inulin was coadministered with psyllium, p=0.0065. Both psyllium and dextrose produced significantly less breath hydrogen than inulin alone, both p<0.0001. Data shown are mean±95% CI.

Psyllium produced no discernible increase in breath hydrogen, whereas breath hydrogen began to rise within 60 min of ingestion of inulin (figure 4). Coadministration of inulin and psyllium both slowed and reduced the maximum rise in breath hydrogen during the study period.

Significant differences were found in AUC breath hydrogen between the test drinks (p<0.0001). Post hoc analysis showed AUC for breath hydrogen after inulin, 7230 (3255–17910) ppm·hour, was significantly greater than after both psyllium (555 (180–915) ppm·hour, p<0.0001) and dextrose (750 (180–1140) ppm·hour, p<0.0001). Coadministration of inulin and psyllium reduced the AUC to 1035 (360–4320) ppm·hour, which was significantly lower than inulin alone, p=0.0065. No differences were found in AUC for breath hydrogen between IBS subtypes for any of the test drinks (online supplemental table S2).

Using the time for breath hydrogen to rise more than 10 ppm from baseline, we can estimate that inulin, when given alone, passed rapidly through the small bowel reaching the caecum within 143±86 min (n=19). Adding psyllium to the inulin slowed this somewhat to 176±86 min (n=8) but the change was not significant, p=0.26. However, it should be noted that in 11 subjects, breath hydrogen after psyllium plus inulin did not rise by 10 ppm and so transit time could not be calculated nor could a transit time for psyllium or dextrose alone. Using MRI detected arrival of fluid in the ascending colon as a marker of orocaecal transit was not possible with inulin as it does not trap fluid and so does not produce a clear increase in signal intensity to mark caecal arrival.

#### Small bowel water content

Test drinks containing psyllium resulted in greater SBWC than the dextrose control. Significant differences were found in SBWC AUC between test drinks (RM ANOVA, p<0.0001); the values with inulin alone (47.2±22.9 L·min) did not significantly differ from those with dextrose (42.4±23.4 L·min, p=0.63) but adding psyllium to inulin caused a significant increase to 86.9±42.8 L·min, p=0.0007. Psyllium alone induced the greatest SBWC 102.7±49.1 L·min, significantly greater than seen with inulin and psyllium coadministration (p=0.03) (online supplemental figure 3). There were however no differences in SBWC between IBS subtype for any of the test drinks (online supplemental table 2).

#### IBS symptoms

Symptoms were typically mild to moderate, the main symptom being flatulence which rose steadily during the study period for all test drinks. Six hours post ingestion there were significant differences between test drinks for flatulence (p*=*0.01), inulin leading to more severe symptoms than psyllium (1.2±0.8 vs 0.5±0.5, p*=*0.04). Coadministration of inulin and psyllium resulted in intermediate values which were not significantly different from inulin alone. Considering all patients with IBS, no differences were found between test drinks for abdominal pain or bloating. However, when subgroups IBS-C and IBS-D were compared, IBS-D showed greater pain and bloating after inulin alone (p=0.04 and p=0.01, respectively) and greater pain after inulin and psyllium coadministration (p=0.03 (online supplemental table S2)).

### In vitro fermentation study

**Figure 5:**
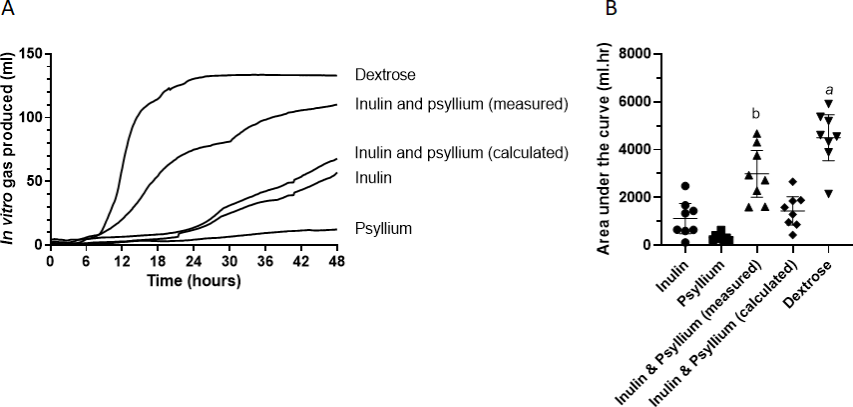
Forty-eight hours of *in vitro* gas production of substrates inoculated using stool from patients who participated in the human MRI study (n=8). (A) Median cumulative gas production showing an early rapid fermentation of dextrose followed by the inulin and psyllium combination. (B) Area under the curve (AUC) of *in vitro* gas production for each test drink (on the x axis). Data shown are mean±95% CI. (A) Dextrose AUC is significantly greater than inulin (p=0.0008), psyllium (p=0.0001) and calculated inulin and psyllium (p=0.001). (B) Inulin and psyllium combination AUC is significantly greater than psyllium (p=0.002).

Bacterial fermentation of all test fibres and the placebo dextrose resulted in the production of gas which was greatest for dextrose (AUC 4500±1144 mL·hour), significantly higher than for inulin alone and psyllium alone (p<0.001 for both (figure 5)).

Of the substrates likely to reach the colon intact, coadministration of inulin and psyllium to the fermentation model generated the most gas (AUC of 2991±1169 mL·hour), whereas inulin alone produced less gas (1122±758 mL·hour), although the difference was not significant, p=0.056. Psyllium alone resulted in the lowest amount of gas production (324±146 mL·hour), which was significantly lower than coadministration of inulin and psyllium (p*=*0.02). The calculated values for inulin and psyllium gas production (the sum of inulin alone plus psyllium alone) gave a lower mean AUC (1446±704 mL·hour) than that actually measured during coadministration, although this difference was not significant (p=0.1).

**Figure 6:**
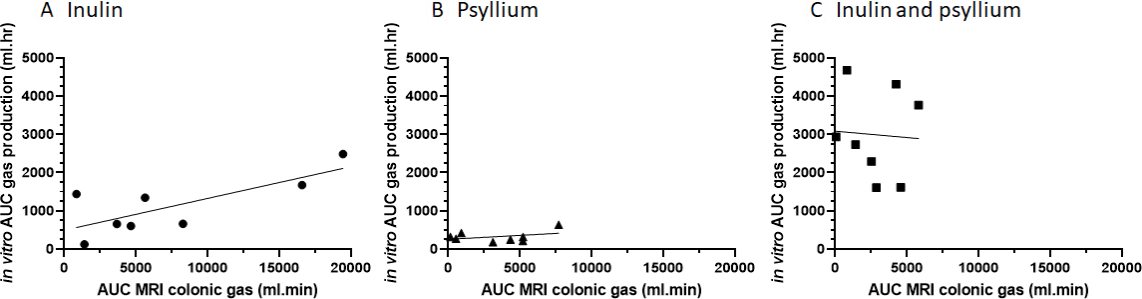
Correlations between *in vitro* area under the curve (AUC) gas production (mL·hour) and *in vivo* AUC colonic gas (mL·min) as assessed by MRI for (A) inulin r^2^=0.58, p=0.03, (B) psyllium r^2^=0.14, p=0.35 and (C) inulin and psyllium in combination r^2^=0.003, p=0.89.

There were no differences in gas production for any fibre between IBS subtypes (online supplemental figure 4). Gas production *in vitro* and *in vivo* were both highly variable but correlated strongly for inulin (r^2^=0.58, p*=*0.03) but not for psyllium (r^2^=0.14, p=0.35) or coadministration of inulin and psyllium (r^2^=0.003, p=0.89 (figure 6)).

#### Viscosity of substrates

Coadministration of inulin to psyllium did not alter the viscosity when compared with psyllium alone. Concentrations used *in vitro* and *in vivo* both resulted in formation of a weak gel as indicated by the storage modulus (solid-like behaviour) (G′) values being higher compared with the loss modulus (liquid-like behaviour) (G″) under the conditions used both *in vivo* and *in vitro* (online supplemental figure S5).

## Discussion

Our study confirms previous data from healthy volunteers and patients with IBS^3 14^ that inulin has little effect in the small bowel but is rapidly fermented in the colon causing a rise in colonic gas and breath hydrogen. Previous studies have shown that psyllium,^11^ wheat bran and prickly pear cactus fibre (nopal)^17^ increase colonic volumes but did not study the impact of coadministered inulin on fermentation. The current study confirmed our hypothesis that both rise in colonic gas and breath hydrogen after inulin can be largely inhibited by concurrent ingestion of psyllium.

We chose inulin as an important example of poorly absorbed dietary polysaccharide commonly found in wheat, onion, banana, garlic, and leeks,^28^ food types often associated with symptoms in patients with IBS.^29^ Food manufacturers seeking health benefit claims have sought to use inulin, a storage fructan polymer easily obtained from chicory root, to increase the fibre content of many processed foods. However, its simple linear structure means that compared with other sources of fibre it is rapidly fermented meaning it often causes symptoms. This is particularly true for patients with IBS in whom distension of the colon by fermentation of inulin generates symptoms not seen with healthy volunteers.^3^

Whether fermentation produces gas depends on complex cross-feeding networks in which some bacteria degrade inulin, while others such as *Bifidobacteria* use the products of degradation to generate short chain fatty acids (SCFAs) including butyrate.^30^ The production of H_2_, CH_4_ and CO_2_ after ingestion of large amounts of inulin reflects its rapid fermentation in an anaerobic environment. It is possible that slowing fermentation would allow more efficient metabolism with transfer of reducing equivalents to acetate and other SCFAs rather than gas. We have previously shown both *in vitro* and *in vivo* that psyllium is poorly fermented and produces a much smaller rise in breath hydrogen than equal doses of more readily fermentable fibres including wheat bran and the prickly pear cactus fibre, nopal.^17^ It follows therefore that adding psyllium to inulin would not be expected to increase the gas burden but why it reduces colonic gas requires further explanation.

Our *in vitro* studies were designed to explore the possibility that combining inulin and psyllium would inhibit fermentation directly. However, this actually enhanced gas production in the model where complete mixing was facilitated by the low viscosity of the concentrations used. Thus, the effect we observed *in vivo* was not due to any direct inhibition of colonic fermentation by psyllium. Furthermore, we showed that the inulin and psyllium combination had the same high viscosity seen with psyllium alone which did not per se inhibit fermentation. We hypothesise therefore that the *in vivo* effect is due to the increase in chyme viscosity leading to both a reduction in the rate of delivery of inulin to the colon together with restriction of mixing of colonic microbiota with the inulin bolus.

This hypothesis is supported in part by our observation in subjects that produced hydrogen, that orocaecal transit time (OCTT) was delayed by around 23% by psyllium. However, this needs further study using a method of measuring OCTT which does not depend on fermentation, since in this study, using the breath hydrogen technique, it was only possible to measure OCTT in 8/19 subjects coadministered psyllium. Considerations of subject comfort and inconvenience meant that our study was limited to 8 hours so we cannot be certain that the total hydrogen production over 12–24 hours might not have been similar. Nevertheless, we can be certain that the rate of fermentation in the first 6 hours was significantly reduced.

When we simulated *in vitro* the concentrations of psyllium likely to occur in the colon we found a high viscosity, 10 Pa·s, which is likely to impede mixing in the colon and thereby delay access of colonic bacteria to the inulin. Previous MRI studies show that chyme passing through the ileocaecal valve, which coming from the small bowel has a low bacterial count, is distributed largely in the middle of the colon.^11^ Tagging studies show that within the haustra, close to the colonic wall, there is much less movement and mixing which may favour anaerobic fermentation.^31^ We hypothesise that psyllium impairs movement from the main stream into the haustral pockets and hence delays fermentation.

It is worth pointing out that MRI gas measurements are based on selecting a minimum of five adjacent voxels with signal intensity below a threshold (defined from an area outside the patient) which occupy volumes greater than 0.1 mL, so will miss smaller, more homogenously dispersed gas. The values therefore are the minimum amount of gas present. The technique measures all gases equally. The large interindividual variation in gas volumes can in part be explained by our observation that large gas pockets can rapidly move through the colonic regions and be expelled, causing large differences in gas volumes seen on MRI from one scan to the next. This is in keeping with earlier studies which used a ventilated tent and a smaller dose of poorly absorbed carbohydrate and showed that a substantial amount of hydrogen was expelled per rectum.^32^

We had intended to measure methane as well as hydrogen but technical failure prevented this. Methane producers do produce less hydrogen when exposed to poorly absorbed carbohydrate,^33^ however they are found in only a third^34^ or a fifth^35^ of study populations, so with our small numbers we would have been unlikely to be able to show this. Furthermore, since each individual acted as their own control it is unlikely to have altered our findings.

While the 20 g dose of inulin we used substantially exceeds the UK average daily intake which is around 4 g, the dose is less than the 25–30 g recommended as daily intake of total dietary fibre so for a mechanistic study it is not unphysiological. Ileostomy^36^ and intubation studies^9^ show that >85% of ingested inulin enters the colon intact where it is largely completely metabolised since very little is excreted in stool.^37^ Inulin supplements in humans selectively increase *Bifidobacterium* and *Anaerostipes*,^38 39^ genera which comprise a quarter of the colonic bacteria and in concert with other bacteria are capable of rapidly fermenting inulin and producing large amounts of SCFAs, especially butyrate which is thought to underlie its potential health benefits. The findings that both inulin and inulin plus psyllium resulted in more gas and pain in IBS-D also support the idea that the faster orocaecal transit, noted in previous MRI studies,^40^ would increase the rate of delivery of substrate to the caecal microbiota. We hypothesise that this would overwhelm the microbiota’s ability to use the excess reducing equivalents generated, which would therefore be disposed of as hydrogen or methane rather than used to generate SCFAs. However, to confirm or refute this hypothesis requires more detailed study.

Our mechanistic study was powered on MRI gas volume and was not adequately powered for symptoms, however the fact that the greatest colonic volume was seen with the psyllium and inulin coadministration with no apparent worsening of symptoms suggests that other factors are important. Previous studies have indicated that increased tone is a more important determinant of pain during distension than volume alone.^41^ Psyllium, by slowing fermentation, reduces not only gas but also other products of fermentation such as SCFAs. These activate enteroendocrine cells and potentially stimulate motility and tone.^42^ Thus, reducing these products may reduce symptoms such as pain despite the greater volume. Future studies which measure both volume and microbial metabolites would help resolve these questions.

The 20 g dose of psyllium we used would be expected to transit through the small intestine largely intact and be distributed within the ascending and transverse colon, a volume of 400–600 mL^27^. The unusually high side-chain density of psyllium limits bacterial access so that it is poorly fermented in the colon compared with other viscous fibres,^43 44^ producing little gas as the current study confirmed. Although time constraints on scanning prevented study of events later in the day, from other studies we can expect the fermentation to continue slowly during transit through the distal colon. Further benefits from delaying fermentation in the proximal colon would include shifting SCFA production and particularly butyrate production more distally, where the highest cancer risk lies. Exactly how this will alter colonic physiology is uncertain but increases in butyrate can stimulate enteric nerve growth^45^ and potentially sensitise the colon.^46^ Whether our psyllium intervention would achieve a big enough change to produce such changes should be further investigated.

Possibly more relevant are the studies which have shown that adding wheat bran fibre reduced carcinogenesis in rats fed resistant starch from raw potato,^47^ thought to be mediated by changes in fermentation though the precise mechanism was unclear. Subsequent studies showed that fermentation of resistant starch in pigs was delayed and shifted distally by coadministration of wheat bran,^48^ a phenomenon confirmed in humans.^49^ Such changes in fermentation throw new light on the epidemiological associations between colorectal cancer and fibre intake and suggest future studies will need to take in account the type of fibre and not just its amount.

While our studies suggest ways of relieving symptoms without restricting prebiotic intake, it is worth pointing out that evidence for a significant health benefit from prebiotics is largely based on the strong epidemiological associations between fibre intake and disease with relatively few direct intervention studies.^50^ These show significant changes in microbiota and their metabolites but more long-term studies will be needed to confirm that these translate into significant health benefits.

The water trapping softens stool leading to increased stool frequency in both healthy and constipated subjects^11^ without however changing transit, despite an increase in colonic volume. This lack of effect on transit is unexplained since distension of the colon by balloon^51^ or osmotic laxatives such as macrogols^52^ stimulates propulsive colonic contractions. A possible explanation is that psyllium dampens the response to distension by reducing access to colonic sensors of luminal stimulants such as bile acids and faecal proteases. A recent study using xyloglucan supports the idea that enhancing the barrier between lumen and mucosa by dietary means has promise in the treatment of IBS.^53^ Further studies are plainly warranted of this simple and safe method of alleviating symptoms in patients with IBS.

## Conclusion

Our small mechanistic study has demonstrated that adding psyllium to inulin reduces gas production in patients with IBS and suggests that by choosing diets with adequate amounts of viscous fibre, patients may be able to obtain the prebiotic health benefits of high-fibre diets without exacerbating their IBS symptoms, particularly flatulence. Larger clinical trials are now indicated to confirm the clinical value of such mechanistic insights.

## Data Availability

Data are held with the University of Nottingham and are available upon reasonable request to RS (robin.spiller@nottingham.ac.uk).
