# Supplementary material for: Psyllium reduces inulin-induced colonic gas production in IBS: MRI and in vitro fermentation studies
Source: Gut. 2021 Aug 5;71(5):919–27. doi: 10.1136/gutjnl-2021-324784 (PMC8995815; doi:10.1136/gutjnl-2021-324784)
Supplement: Supplementary data [file gutjnl-2021-324784supp001.pdf]

1

SUPPLEMENTAL MATERIAL

Supplemental Tables

Supplemental Table S1a: Nutritional composition of standardised meal consumed the evening prior to MRI scans

	Energy (kcal)	Carbohydrate (g)	Protein (g)	Fat (g)	Fibre (g)
Lean corned beef (Princes) 200g	194	1.0	25	10	-
Half of 300g tinned whole carrots in water (Sainsbury's)	25	4.3	0.5	0.3	1.9
Steamed basmati plain rice (Tilda) 250g	358	70.4	7.2	4.8	1.8
2 Highland All Butter Shortbread finger biscuits (Sainsbury's)	208	23.8	2	11.6	1
Total kcal= 950.6	785	398	138.8	240.3	9.4

Supplemental Table S1b: Nutritional information of meals consumed during the study day

	Energy (kcal)	Carbohydrate (g)	Protein (g)	Fat (g)	Fibre (g)
220 g Sainsbury's creamed rice pudding	211	37	6.8	3.7	<0.5
34g Sainsbury's seedless raspberry jam	85	21	<0.5	<0.5	0.9
100 mL Sainsbury's pure orange juice from concentrate	42	8.6	0.6	<0.5	<0.5
Total calories	338	266.4	29.6	33.3	1.8

Supplemental Table S2: Exploratory analysis of endpoints divided by IBS subtype. P value relates to testing for differences between the subtypes using Mann-Whitney tests or unpaired t-tests as appropriate. AUC, area under the curve; IBS-C, constipation-predominant irritable bowel syndrome; IBS-D, diarrhoea-predominant irritable bowel syndrome.

	**Test drink**	**IBS-C**	**IBS-D**	***p* value**
MRI colonic gas change from fasting AUC (ml.min)	Inulin, median (IQR)	1080 (282-3717)	5728 (2172-16772)	0.01
	Psyllium, median (IQR)	-12 (-429-1255)	1402 (-660-4418)	0.24
	Inulin and Psyllium, median (IQR)	212 (-1400-1063)	2057 (198-6742)	0.03
	Dextrose, mean±SD	416±1186	3063±4750	0.12
Colonic volume AUC (L.min)	Inulin, mean±SD	319.1±88.9	375.8±76.2	0.15
	Psyllium, mean±SD	296.5±105.7	376.4±72.5	0.08
	Inulin and psyllium, mean±SD	330.6±108.3	384.2±105.1	0.28
	Dextrose, mean±SD	275.5±80.3	322.6±56.4	0.15
Small bowel water content AUC (l.min)	Inulin, mean±SD	46.7±28.2	47.6±18.6	0.45
	Psyllium, mean±SD	112.3±55.9	94.0±43.2	0.43
	Inulin and Psyllium, mean±SD	94.2±50.0	80.2±36.5	0.32
	Dextrose, mean±SD	41.9±32.7	42.8±12.3	0.94
Breath hydrogen AUC (ppm.hr)	Inulin, mean±SD	12432±9354	12623±12331	0.97
	Psyllium, median (IQR)	900 (390-1080)	615 (300-949)	0.33
	Inulin and Psyllium, median (IQR)	1290 (510-6330)	1133 (338-4913)	0.62
	Dextrose, median (IQR)	1065 (653-2055)	608 (41-1958)	0.32
Flatulence score AUC (arbitrary unit.min)	Inulin, median (IQR)	150 (79-443)	281 (203-773)	0.16
	Psyllium, median (IQR)	135 (64-315)	206 (62-319)	0.92
	Inulin and Psyllium, median (IQR)	105 (41-341)	270 (113-465)	0.29
	Dextrose, mean±SD	253±278	406±364	0.32
Bloating score AUC (arbitrary unit.min)	Inulin, median (IQR)	15 (8-420)	379 (219-958)	0.04
	Psyllium, mean±SD	262±268	407±321	0.30
	Inulin and Psyllium, median (IQR)	173 (49-338)	450 (191-840)	0.08
	Dextrose, median (IQR)	98 (0-236)	278 (81-435)	0.11
	Inulin, median (IQR)	30 (0-64)	184 (98-939)	0.01

Abdominal pain score AUC (arbitrary unit.min)	Psyllium, median (IQR)	38 (8-184)	218 (43-572)	0.09
	Inulin and Psyllium, median (IQR)	0 (0-143)	289 (73-600)	0.03
	Dextrose, median (IQR)	0 (0-53)	120 (6-373)	0.14

Supplementary Table S3: Area under the curve of colon volumes (in L.min, mean ± SD) for the ascending, transverse, descending and sigmoid colon for each test drink. P value relates to testing for differences using analysis of variance between the test drinks for each region of the colon.

	**Inulin**	**Inulin and Psyllium**	**Psyllium**	**Dextrose**	**P value**
Ascending colon AUC (L.min)	121±32[a]	134±42 [c]	124±38	107±30	0.0004
Transverse colon AUC (L.min)	117±34[a]	124±51[a]	106±40	100±35	0.03
Descending colon AUC (L.min)	68±26	66±32	68±27	59±20	0.2
Sigmoid colon AUC (L.min)	44±24[b]	38±17	41±20	34±17	0.05

[a] significantly greater than dextrose, p<0.05
[b] significantly greater than dextrose, p<0.005
[c] significantly greater than dextrose, p<0.0005

MRI endpoints and methods

MRI data analysists were blinded to the intervention received. A range of MRI sequences were used to image the abdomen to obtain the various endpoints including:

1) Colonic gas was assessed as previously published[1] using a dual echo gradient echo sequence (TR 175 ms, TE1= 2ms, TE2 = 4.3 ms, FA 80°, ASSET 2) to acquire 24 coronal images with a slice thickness 7mm (no gap) and reconstructed in plane resolution of 1.76 x 1.76 mm^2. This sequence was used to measure colonic volumes as well and was acquired during a breathhold. An additional identical sequence was also acquired with the R.F. power set to zero to acquire the noise distribution across the images for gas measurements.
2) Small bowel water content was measured as previously reported[2] using a single shot fast spin echo (SSFSE) sequence with fat saturation (TE_{eff} = 325 ms, Echo spacing 5ms) to acquire 32 coronal images with a 7mm slice thickness (no gap) and reconstructed in plane resolution of 0.78 x 0.78 mm^2.
3) Colonic volumes were measured from the dual echo images as previously reported[3] using MIPAV software[4] to segment the different colonic regions.

In vitro fermentation study details

Gas production from the fermentation of the test substrates was measured using the ANKOM RF gas production system (ANKOM Technology, Macedon, NY, USA). Briefly, per 125ml bottle, 0.5g of psyllium, inulin, or dextrose were added to each fermentation bottle. Additionally, 0.5g of psyllium and 0.5g of inulin were also added to bottles to test the impact of the substrates combined on gas production (inulin + psyllium). To remove any discrepancies in gas produced from sources other than the test substrates, a non-substrate blank was used. To each fermentation bottle, 76ml of media, 5ml of a vitamin and phosphate buffer solution, and 1ml of the reducing solution[5] were added under a constant stream of CO_2. Once sealed, the substrates were allowed to hydrate, and bottles pre-warmed overnight at 37°C.

Bottles were seeded with faecal samples from eight of the IBS individuals from the human MRI study (four IBS-C, four IBS-D). Faecal samples were frozen at -80°C, therefore prior to testing, each faecal sample was defrosted at room temperature. Once defrosted they were diluted in pre-reduced PBS (10% wt/vol), homogenised in a stomacher and strained to remove particulates. Each substrate was fermented in triplicate per volunteer faecal sample. Each bottle was inoculated with 3ml of slurry, sealed, and incubated at 37°C in a shaking water bath (80 rpm) for five days.

Using the ANKOM RF system, the gas pressure was automatically measured every 15 minutes. Gas production from fibre was calculated using previous methods[6]. Data are reported as cumulative gas volume produced during fermentation, averaged from eight IBS individuals and measured in triplicate per individual/substrate type, thus a total of 24 individual fermentations were performed per substrate.

5

Stool collection instructions

1. Use the complete kit provided. It contains:
   - Cardboard tray
   - 1 Plastic sample tube with spoon attached to lid
   - Larger opaque plastic bag
   - 1 pair rubber gloves
   - Ice/frozen gel packs
   - Cooler bag/container

2. If possible, urinate before stool collection to avoid mixing urine and stool as this may affect the sample.

   THEN PLEASE WASH HANDS YOUR HANDS

3. Place or hold the cardboard tray to collect the stool as passed.

   THEN PLEASE WASH HANDS YOUR HANDS

4. After opening your bowels, put on the gloves provided (ensure your hands are dry or these will be difficult to put on).

5. Place a large amount of the stool passed into the tube so that it is at least 75% full. To do this, use the spoon attached to the tube as seen in the image below.

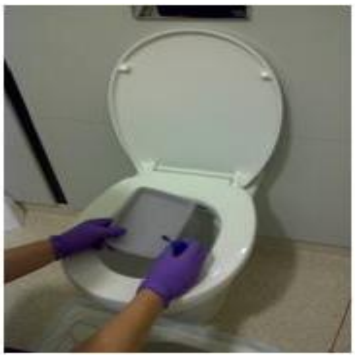

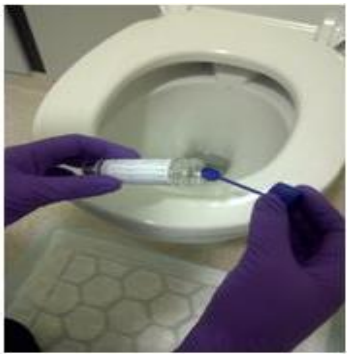

6. Place the spoon and stool sample into the tube and lightly secure the lid. **DO NOT SCREW THE TOP ON TIGHTLY.**

7. After stool collection the remaining stool sample should be flushed away. The rubber gloves and cardboard tray should be placed in a plastic bag and disposed of in your bin as normal rubbish.

8. Place the tube with stool samples into the transparent plastic bag and seal the bag tightly.

9. Place the transparent plastic bag and the ice packs inside the cooler bag/ container provided. This should be sealed and placed in your freezer.

10. Ideally the stool sample should be returned to us as soon as possible (within a few days). Please keep the sample frozen. When you are ready to take the sample to the research site, the cooler bag can be removed from the freezer and taken to your next appointment.

We appreciate that some participants may not have access to a freezer in your own home, and if this is the case please inform us so we can make alternative arrangements

Supplemental figures

Supplemental Figure S1

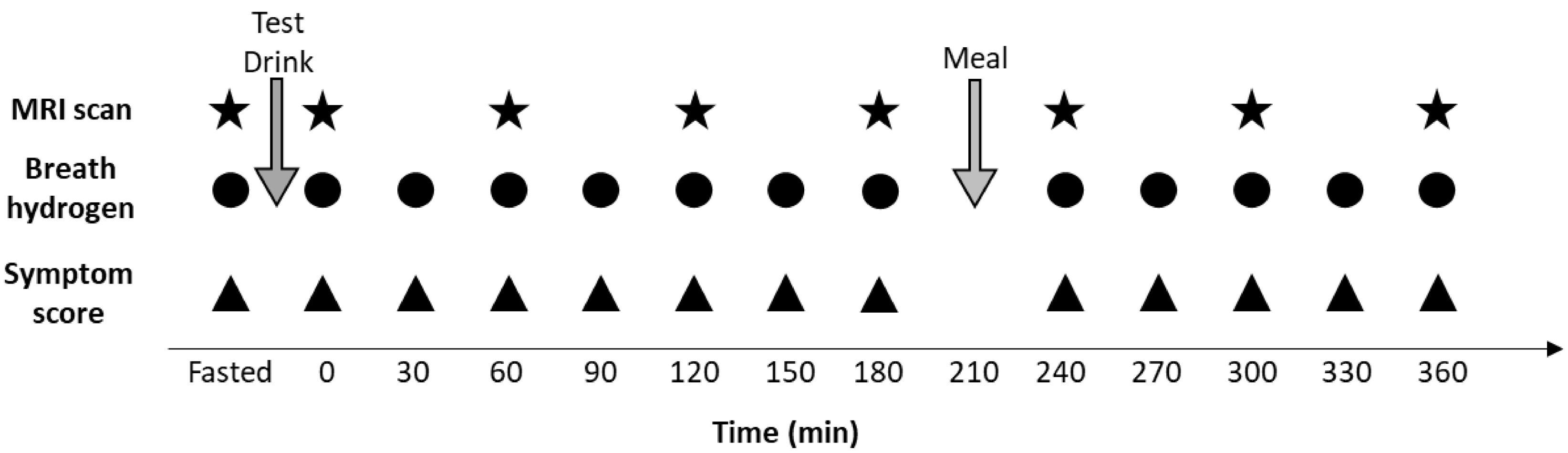

Supplemental figure S1: Schematic of the study day. MRI scans were performed fasted, immediately after the test drink and at hourly intervals while breath hydrogen and symptom scores were obtained at 30 minute intervals. Test drinks were consumed before time 0 scan and the standardised meal given at 210 minutes.

Supplemental Figure S2

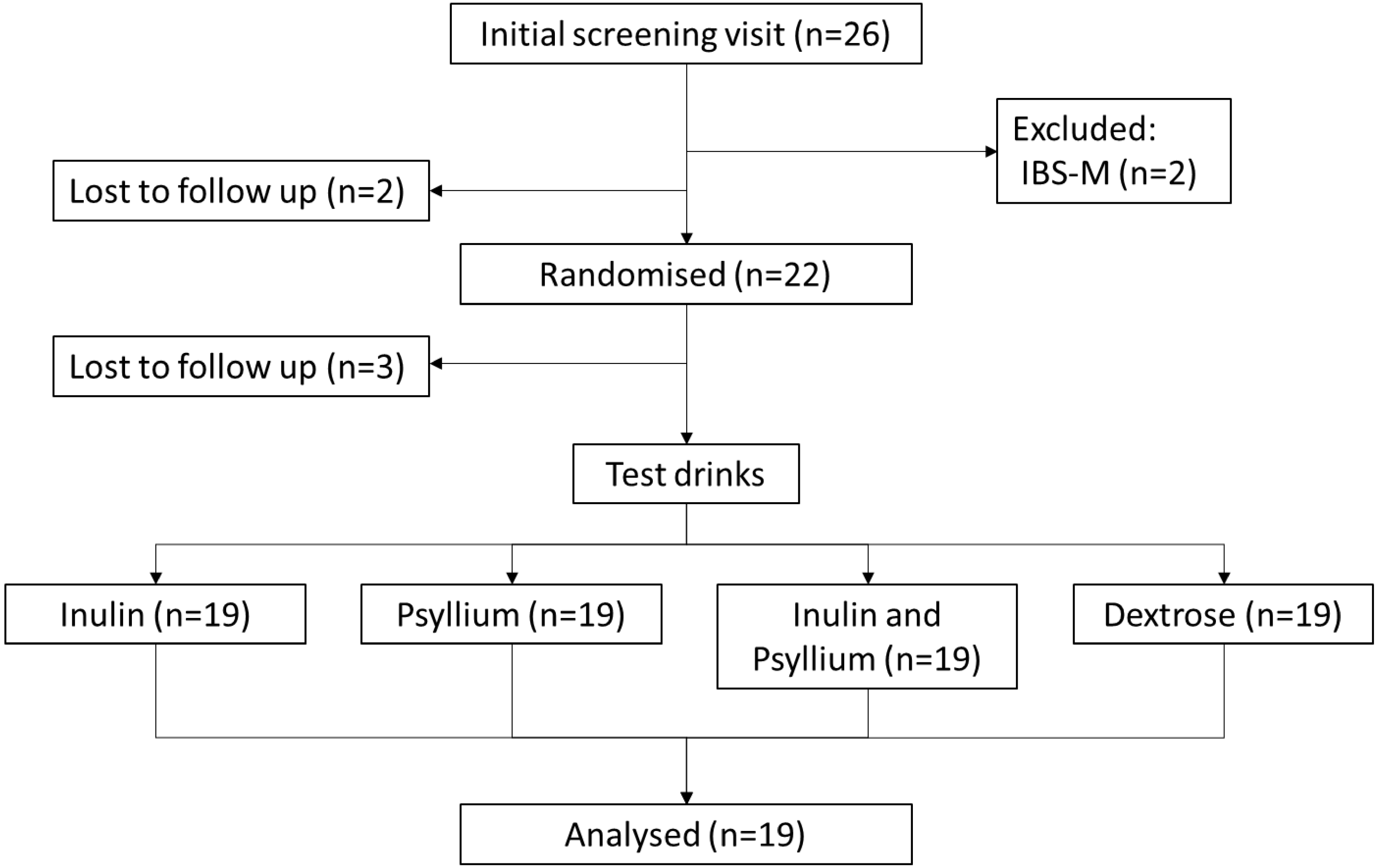

Supplemental Figure S2: CONSORT diagram of the study. IBS-M, Mixed-type irritable bowel syndrome.

Supplemental Figure S3

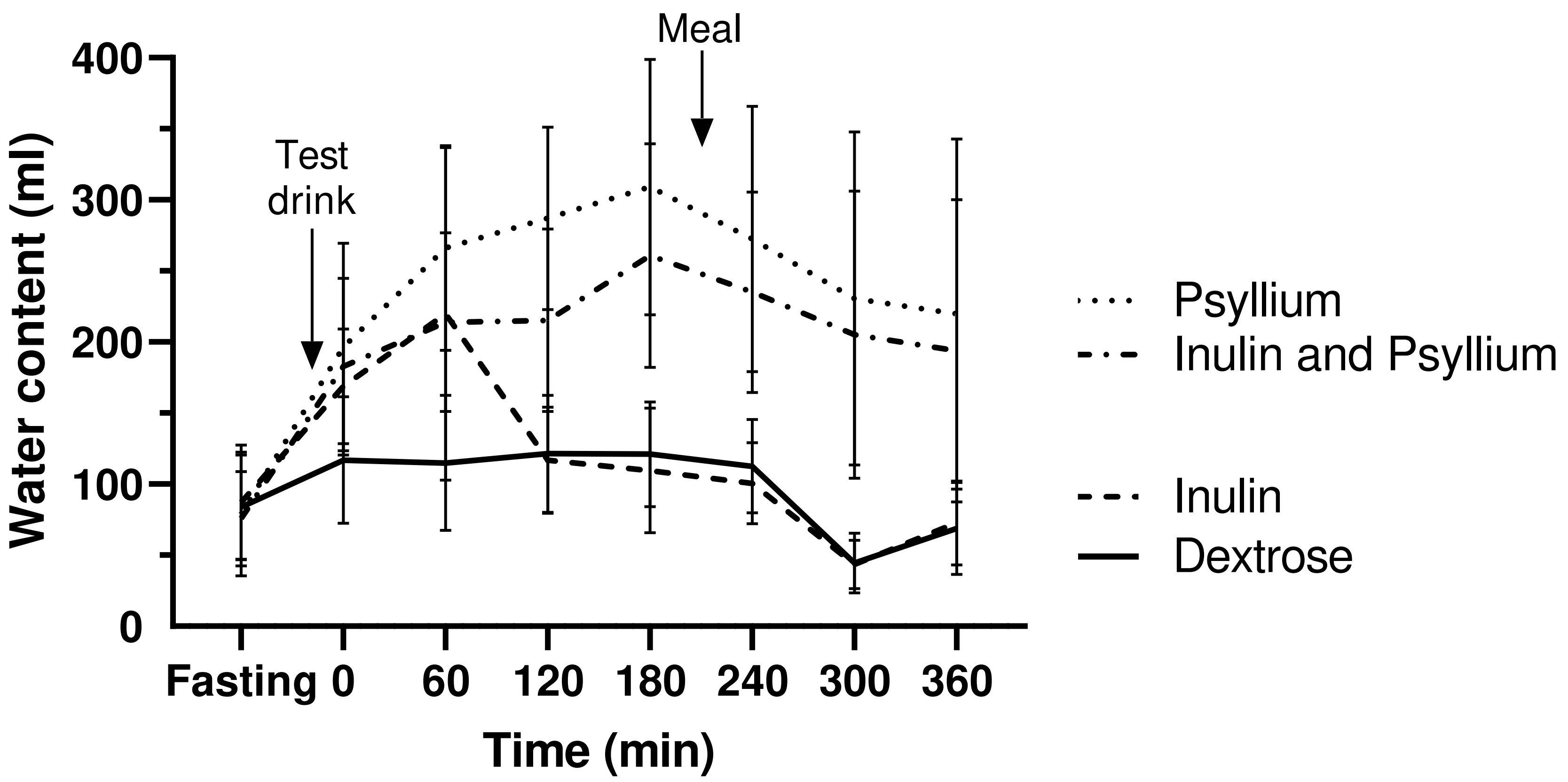

Supplemental Figure S3: Small bowel water content (SBWC) rose after test drinks containing psyllium but hardly at all after dextrose. Comparison of areas under the curve (AUCs) showed psyllium was associated with the highest values, significantly greater than inulin plus

psyllium, *p*=0.03. Adding psyllium to inulin produced a significant rise in SBWC, *p*=0.0007. Inulin and dextrose were not significantly different. Data shown are mean ± 95% CI.

11

Supplemental Figure S4

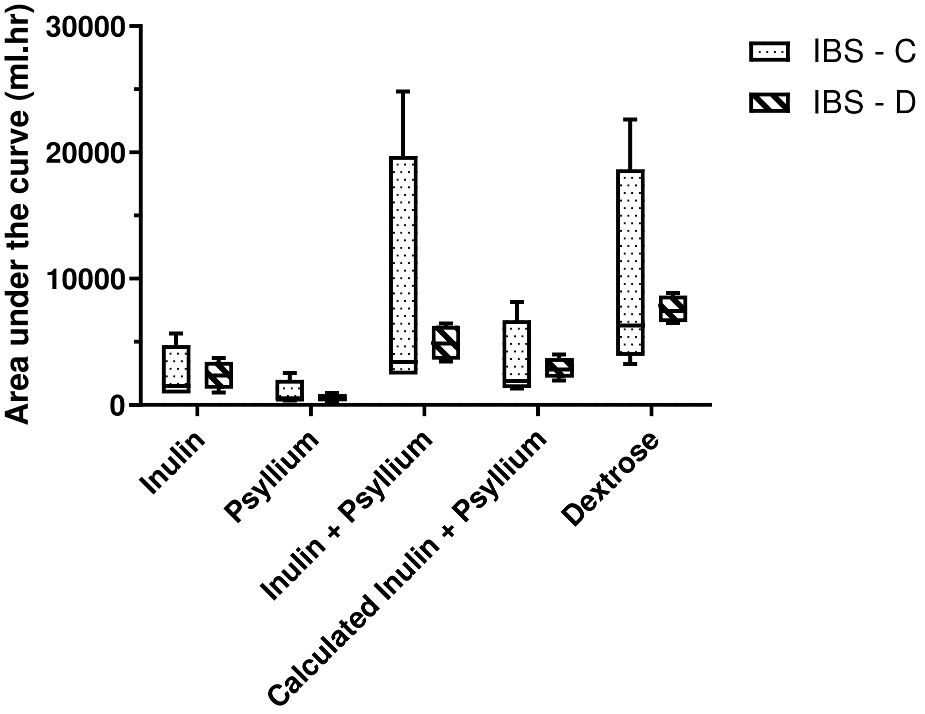

Supplemental Figure S4: Tukey box and whiskers plot of area under the curve (ml.hr) after 48 hours of *in vitro* gas production, divided by IBS subtype (n=4 for each). No significant differences were found between subtypes. IBS-C, constipation predominant irritable bowel syndrome; IBS-D, diarrhoea predominant irritable bowel syndrome.

12

Supplemental Figure S5

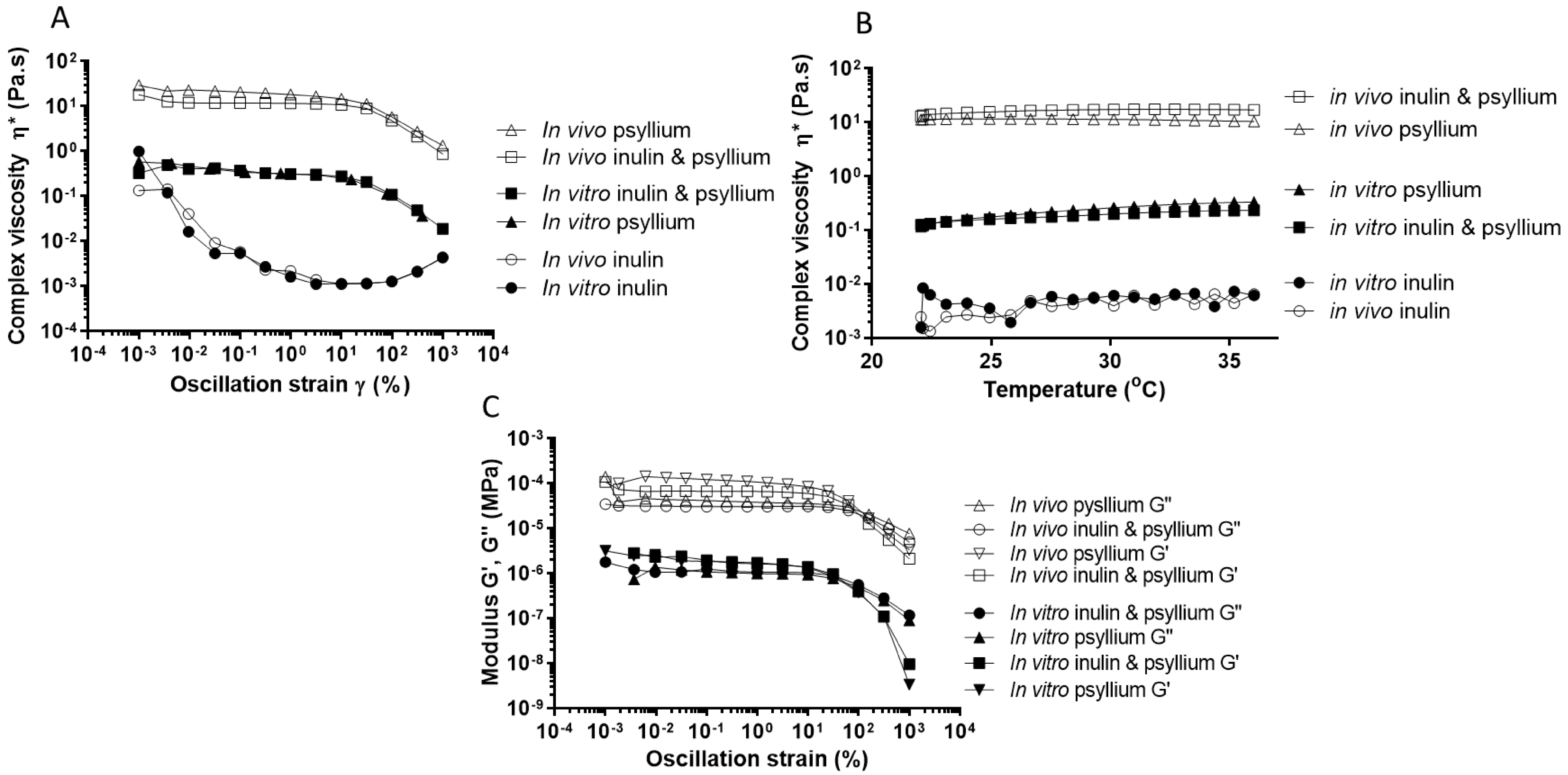

Supplemental Figure S5: Rheometric analysis of substrates tested. Samples labelled *in vitro* were 0.5% solution while those labelled *in vivo* were 4% solution, this being the estimated concentration *in vivo* assuming 20g of ingested psyllium is diluted in 500ml of colonic water, colonic

volumes being based on MRI assessment by Pritchard *et al*[3]. Analysis was performed with the AR-G2 magnetic bearing rheometer (TA instruments) with a cup (diameter: 30 mm) and vane (bob diameter 28 mm). Inulin was prepared by solubilising in boiling water and storing at 4°C overnight. Psyllium was mixed with the water or the inulin solution immediately prior to analysis. Parameters for data within oscillation strain sweeps ranging from 10^{-3} to 10^{3} at 37°C and a frequency of 6.28 rad/s (A, C).

Data shows that the addition of inulin to the psyllium did not alter the viscosity compared to psyllium alone (A). Varying the temperature from room to body temperature did not affect results (B). The 4% solution was more viscous than the 0.5% solution, but both solutions yielded gel structures as demonstrated by an increase in G' (storage modulus) compared to the G" (loss modulus). These gels also behaved the same whereby they lost their structure, becoming a liquid at the same oscillation strain (C).

REFERENCES

1. Coletta M, Gates FK, Marciani L, et al. Effect of bread gluten content on gastrointestinal function: A crossover MRI study on healthy humans. *Br J Nutr*. 2016;115(1):55-61. doi:10.1017/S0007114515004183

2. Hoad CL, Marciani L, Foley S, et al. Non-invasive quantification of small bowel water content by MRI: a validation study. *Phys Med Biol*. 2007;52(23):6909-6922. doi:10.1088/0031-9155/52/23/009

3. Pritchard SE, Marciani L, Garsed KC, et al. Fasting and postprandial volumes of the undisturbed colon: Normal values and changes in diarrhea-predominant irritable bowel syndrome measured using serial MRI. *Neurogastroenterol Motil*. 2014;26(1):124-130. doi:10.1111/nmo.12243

4. McAuliffe MJ, Lalonde FM, McGarry D, Gandler W, Csaky K, Trus BL. Medical image processing, analysis & visualization in clinical research. In: *Proceedings of the IEEE Symposium on Computer-Based Medical Systems*. ; 2001:381-388. doi:10.1109/cbms.2001.941749

5. Williams BA, Bosch MW, Boer H, Verstegen MWA, Tamminga S. An in vitro batch culture method to assess potential fermentability of feed ingredients for monogastric diets. *Anim Feed Sci Technol*. 2005;123-124 Pa:445-462. doi:10.1016/j.anifeedsci.2005.04.031

6. Rotbart A, Yao CK, Ha N, et al. Designing an in-vitro gas profiling system for human faecal samples. *Sensors Actuators, B Chem*. 2017;238:754-764. doi:10.1016/j.snb.2016.07.120
